# Antibiotic Resistance, and Biofilm Forming Characteristics of Escherichia coli Clinical Isolates at a Hospital in Tigray, Northern Ethiopia

**DOI:** 10.7759/cureus.73569

**Published:** 2024-11-13

**Authors:** Teklay Gebremariam, Tadesse Eguale, Tesfaye Belay, Alem A Kalayu, Teferra Abula, Ephrem Engidawork

**Affiliations:** 1 Department of Pharmacology, School of Pharmacy, College of Health Sciences, Addis Ababa University, Addis Ababa, ETH; 2 Microbiology Unit, Aklilu Lemma Institute of Pathobiology, Addis Ababa University, Addis Ababa, ETH; 3 Department of Applied Sciences and Mathematics, School of Stem, Blue Field State College, Bluefield, USA; 4 Department of Microbiology, Immunology and Parasitology, School of Medicine, College of Health Sciences, Addis Ababa University, Addis Ababa, ETH

**Keywords:** ampicillinc beta-lactamase, biofilm, escherichia coli, extended-spectrum beta-lactamase, tissue culture plate method

## Abstract

Background: *Escherichia coli (E. coli)* infections are becoming difficult to treat due to the bacterium's biofilm-forming capabilities and rising resistance to multiple antibiotics, posing a growing clinical challenge. This study assessed the antimicrobial resistance and biofilm formation by *Escherichia coli* isolates from patients at a hospital in Tigray, Northern Ethiopia.

Method: From patients exhibiting signs of bacterial infection, while excluding recent antibiotic users or those with incomplete data, 417 clinical samples comprised of 84 blood, 108 pus, and 225 urine samples were obtained in a cross-sectional study. The combination disc method was used to test extended-spectrum beta-lactamase (ESBL) production, and Ampicillin C (AmpC) enzyme presence was confirmed with cefoxitin and cefotaxime discs. Data analysis was conducted with SPSS version 22 software, applying ANOVA and logistic regression, with significance set at p<0.05.

Result: Among the 417 samples, 109 (26.1%) tested positive for *Escherichia coli*. These isolates showed high resistance to ampicillin (84.4%) but lower resistance to meropenem (9.17%). ESBL was detected in 46.8% of isolates and AmpC in 54%, with 48 (44%) isolates positive for both. Strong biofilm formation occurred in 76% of isolates, while only 2.75% were weak producers. Biofilm strength correlated significantly with prior antibiotic use (p=0.028), ward type (p=0.001), and use of indwelling devices (p=0.000).

Conclusion: In northern Ethiopia, *Escherichia coli* isolates showed resistance to major antibiotic classes like beta-lactams, fluoroquinolones, and aminoglycosides. This high resistance and biofilm development highlight the critical need for interventions to curb resistance spread, with a focus on antibiofilm research and enhanced infection prevention measures.

## Introduction

*Escherichia coli (E. coli)*, a gram-negative bacterium in the Enterobacteriaceae family, is a leading pathogen in healthcare environments, responsible for a variety of infections, such as urinary tract infections (UTIs), hospital-acquired bloodstream infections, surgical site infections, and gastrointestinal infections [1,2]. The growing issue of antimicrobial resistance (AMR) and biofilm formation in *E. coli* adds complexity to treatment, increasing morbidity, hospital stays, and healthcare costs. Effective management of *E. coli* infections is essential to improve patient outcomes and limit the spread of resistant strains [1-3].

Antimicrobial agents are essential in lowering morbidity and mortality associated with infectious diseases [4,5]. Research across various African regions has documented a rising trend in antimicrobial resistance among enteric bacteria, including *E. coli*, indicating a growing challenge in managing these infections effectively [5,6].

The occurrence of antimicrobial resistance (AMR) among bacteria isolated from patients, within hospitals, and from surrounding environments is influenced by multiple factors, such as the frequency of empirical antimicrobial use, treatment duration, patient comorbidities, and the use of indwelling medical devices [6,7]. The growing resistance of *E. coli* to various antimicrobials is significantly linked to its strong biofilm-forming ability. Biofilms increase bacterial virulence, with biofilm-associated infections being 10-1000 times more resistant to treatment than free-floating (planktonic) forms, posing serious challenges to therapy. This biofilm-driven resistance calls for innovative approaches to manage persistent infections effectively [8].

In Tigray, northern Ethiopia, there is limited data on biofilm formation and antimicrobial resistance (AMR) patterns in clinical isolates, which can make it challenging for healthcare providers to make informed treatment decisions. This study aimed to fill this gap by assessing the antimicrobial resistance profile and biofilm-forming characteristics of *E. coli* isolates at Ayder Referral Hospital (ARH) in Tigray, Northern Ethiopia, providing valuable insights to support prescribers.

## Materials and methods

Study area and design

This cross-sectional study was conducted using convenient sampling from December 26, 2019, to December 20, 2020, at ARH in Tigray, north Ethiopia. ARH is the region's largest referral hospital with a 500-bed capacity and serves approximately 3.5 million patients annually, including those from northern Ethiopia and parts of Eritrea. A hospital-based cross-sectional design was employed, recruiting inpatients and outpatients with diverse infection histories, ages, and genders.

Sampling and eligibility

The total sample size was 417, which was calculated using a single population proportion formula, assuming a prevalence of 50% to maximize the sample size, precision of 5%, 95% confidence interval, and 9% nonresponse rate. A total of 417 nonduplicate specimens were collected from all the study participants across various wards. Inclusion criteria comprised patients with complete records, regardless of age or sex, who were willing to participate. To ensure data quality and minimize duplication, the first bacterial isolate from each patient during their admission period was collected, with only one specimen taken per patient with no repeat. A total of 417 specimens were collected, comprising 84 blood samples, 108 pus samples, and 225 urine samples from various wards (medical ward (32 blood, 29 pus, 122 urine), outpatient department (17 blood, 17 pus, 71 urine), pediatric ward (29 blood, 14 pus, 23 urine), and surgical ward (6 blood, 48 pus, 9 urine)).

Specimen collection and processing

Specimens were collected aseptically in sterile, leak-proof containers, labeled appropriately, and processed within an hour of collection at the microbiology laboratory of ARH. Blood samples (10 mL for adults, 1-3 mL for children) were collected in blood culture bottles with brain-heart infusion broth after thorough site disinfection, labeled, and immediately transported for timely processing. Urine (10-20 mL) was collected via midstream clean-catch in sterile containers, labeled, and processed within an hour. Pus samples were aseptically collected using sterile swabs or syringes, with the wound area disinfected beforehand (if closed like a pustule) or cleaned using normal saline (if open wound). All specimens were securely labeled and promptly transported for accurate culture analysis.

Isolation of *E. coli* was performed by direct plating of specimens (pus and urine) or broth from blood culture bottles containing brain-heart infusion (BHI) broth (Oxoid Ltd, UK) onto eosin methylene blue (EMB) agar plates (Himedia Laboratories, India)[9-10]. Each plate was inoculated by streaking with a sterile loop to obtain isolated colonies and then incubated at 35°C for 18 hours. Colonies displaying a characteristic green-metallic sheen were presumptively identified as *E. coli *and confirmed through biochemical tests.

For blood cultures, 5-10 mL of blood was inoculated directly into BHI broth, mixed gently to prevent clotting, and incubated for up to seven days before sub-culturing on EMB agar. All confirmed pure isolates underwent antimicrobial susceptibility testing and biofilm formation assays.

Antimicrobial susceptibility testing

The antimicrobial susceptibility of *E. coli* isolates was assessed against a panel of 14 antimicrobials using the Kirby-Bauer disc diffusion method, following the CLSI guideline [11]. The antimicrobials tested included ampicillin (10 μg), amoxicillin-clavulanate (20/10 μg), cefotaxime (30 μg), cefoxitin (30 μg), ceftazidime (30 μg), trimethoprim-sulfamethoxazole (1.25/23.5 μg), meropenem (10 μg), gentamicin (10 μg), tobramycin (10 μg), amikacin (30 μg), erythromycin (15 μg), tetracycline (30 μg), ciprofloxacin (5 μg), chloramphenicol (30 μg), and nitrofurantoin (300 μg). All antimicrobial discs were obtained from Thermo Scientific™ and Oxoid™, UK. The CLSI cut-off points were applied to categorize the isolates as susceptible, intermediate, or resistant.

Multiple antibiotic resistance index

Isolates were considered as MDR if resistant to three or more antimicrobial classes, including beta-lactams, aminoglycosides, and fluoroquinolones for *E. coli*. XDR isolates showed resistance to all but one or two drug classes, leaving few treatment options. PDR isolates were resistant to all tested antimicrobials, posing substantial therapeutic difficulties due to a lack of effective drugs. *E. coli* (ATCC 25922) served as the reference strain for quality control [10-11].

Multiple antibiotic resistance (MAR) indices were calculated using the formula:

MAR Index=a/b

Where 'a' represents the number of antibiotics to which the isolate is resistant, and 'b' is the total number of antibiotics tested against the isolate [10,12].

Determination of ESBL and AmpC beta-lactamase production

The *E. coli* isolates were tested for extended-spectrum beta-lactamase (ESBL) production by the double-disk synergy test (DDST) using ceftazidime (30 µg), cefotaxime (30 µg), ceftazidime-clavulanate (30/10 µg), and cefotaxime-clavulanate (30/10 µg) discs. The ceftazidime and cefotaxime discs were placed 15 mm apart from their corresponding antimicrobial agent in combination with clavulanate on Muller Hinton agar that was incubated at 35±2^o^C for 16-18 hours. *E. coli* was considered as ESBL-producing if there was a ≥5 mm increase in the zone diameter for either antimicrobial agent in combination with clavulanate versus the zone diameter of the corresponding disc alone [11].

For AmpC beta-lactamase detection, a cefoxitin disk (30 μg) was employed, with further confirmation via the cefoxitin-cloxacillin double-disk synergy test. A positive result for AmpC production was determined by observing a zone diameter difference of 4 mm or more between the cefoxitin/cloxacillin disk and the cefoxitin-only disk [10,13].

Biofilm formation

Biofilm formation was evaluated using the tissue culture plate (TCP) method. A loopful of *E. coli* colonies from an overnight nutrient agar culture was inoculated into 10 mL of trypticase soy broth (TSB) supplemented with 1% glucose [14-15]. The broth was incubated at 37°C for 24 hours, after which the culture was diluted 1:100 with fresh medium. A volume of 0.2 mL of the diluted cultures was dispensed into individual wells of sterile, flat-bottomed 96-well polystyrene TCPs. Sterile broth served as a blank, and control organisms were similarly diluted and incubated [16-17]. All wells, including controls and blanks, were incubated at 37°C for 24 hours. Following incubation, the wells were washed four times with 200 μL of phosphate-buffered saline (PBS, pH 7.2) to remove non-adherent bacteria. Biofilms remaining attached to the well walls and bottoms were fixed with 2% sodium acetate and stained with 0.1% crystal violet. After 30 minutes, the excess stain was removed by washing with deionized water, and the plates were allowed to dry completely.

The optical density (OD) of the stained biofilm was measured at 620 nm using a spectrophotometer. To account for background staining, the average OD of the sterile medium was subtracted from all test values. The OD cutoff value (ODc) was determined by adding the average OD of the negative control to three times the standard deviation (SD) of the negative control. *E. coli *isolates were classified as follows: no biofilm producers (OD≤ODc), weak biofilm producers (ODc<OD≤ 2×ODc), moderate biofilm producers (2×ODc<OD≤4×ODc), and strong biofilm producers (OD>4×ODc). Each experiment was performed independently at least three times [16-17].

Statistical analysis

Data were compiled in Excel and analyzed using IBM SPSS Version 22. Descriptive statistics (percentages, means, standard deviations) were used to summarize the data. Chi-square tests were used to assess associations between factors and antimicrobial resistance (AMR) or biofilm strength, with goodness-of-fit tests evaluating the distribution of categorical variables like antibiotic resistance. A binary logistic regression model was applied to determine the probability of resistance to specific antibiotics, while one-way ANOVA was used to compare means across groups, particularly for biofilm formation. Statistical significance was set at p≤0.05.

Ethical considerations

Ethical approval was obtained from the Institutional Review Board of the College of Health Sciences, Addis Ababa University (Protocol number 033/19/SoP and minute number 05/2019). Data collection was completed only after written informed consent from patients, participants, parents, and/or legal guardians. Assent was also obtained from older children (12-18 years old).

## Results

Sociodemographic and clinical characteristics

The mean age of study participants was 41 years (SD, 21.4). Urine constituted about half (225, 54%) of the specimens collected. About 195 (46.8%) participants had taken antimicrobials as empirical therapy, 140 (33.6%) had different morbidities, and 179 (42.9%) had indwelling medical devices (Table 1). Indwelling devices, such as urinary catheters and intravenous lines, were used, which can promote biofilm growth, increasing infection risk. Samples from these devices were collected aseptically to avoid contamination. Common health conditions like diabetes, hypertension, and respiratory disorders have heightened infection susceptibility. Sample collection from these patients involved extra care, including thorough medication histories, to consider antimicrobials' effects on biofilms.

**Table 1:** Sociodemographic profile and ward allocation of participants at a hospital in Tigray, Northern Ethiopia MW: medical ward; OPD: outpatient department; PW: pediatric ward; SW: surgical ward

Variables	Number of specimens collected	Ward			
		MW	OPD	PW	SW
Age ranges					
<18	59 (14.1%)	7	5	43	4
19-39	169 (40.5%)	79	34	23	33
40-59	109 (26.1%)	56	37	0	16
>60	80 (19.2%)	41	29	0	10
Total	417	183	105	66	63
Sex					
Male	193 (46.28%)	85	47	29	32
Female	224 (53.71%)	98	58	37	31
Total	417	183	105	66	63
Use of indwelling medical devices	179 (42.93%)	102	16	24	37
Intravenous line	55 {13.18}	31	2	9	13
Urinary catheter	90 (21.5)	51	14	7	16
Other devices	34 (8.15)	20	0	6	8
No use of indwelling medical devices	238 (57.07%)	81	89	42	26
Total	417	183	105	66	63
History of antimicrobial use					
Yes	195 (46.76%)	100	28	38	29
No	222 (53.23%)	83	77	28	34
Total	417	183	105	66	63
Presence of comorbidities	140 (33.57%)	73	22	17	28
Infectious	32 {7.67}	14	3	12	3
Endocrine	76 (18.22)	31	15	5	25
Neurologic	14 (3.35)	12	2	0	0
Others	18 (4.31)	16	2	0	0
No presence of comorbidities	277 (66.43%)	110	83	49	35
Total	417	183	105	66	63
Length of hospitalization in days					
0	112 (26.86%)	5	105	1	1
1-7	167 (40.04%)	102	-	27	28
>8	138 (33.09%)	76	-	28	34
Total	417	183	105	66	63
Type of specimen					
Blood	84 (20.14%)	32	17	29	6
Pus	108 (25.9%)	29	17	14	48
Urine	225 (53.96%)	122	71	23	9
Total	417	183	105	66	63

*Escherichia coli *isolation

*E. coli* was isolated from 109 (26.1%) of the 417 clinical samples collected from hospitalized patients exhibiting signs of bacterial infection. The prevalence of *E. coli* varied by hospital ward, with the highest occurrence noted in the medical ward (49, 45%) and the lowest in the surgical ward (16, 14.7%) (Table 2). In terms of specimen types, *E. coli* was most commonly isolated from urine samples (67, 61.5%), followed by pus (28, 25.7%) and blood (14, 12.84%). Significantly higher isolation rates of pathogenic *E. coli* were linked to certain patient demographics: individuals with comorbidities (67, 61.5%; p=0.005), those with no recent history of antimicrobial use (56, 51.4%; p=0.000), and patients who had been hospitalized for more than eight days (66, 60.6%; p=0.000) (Table 2).

**Table 2:** Clinical profile and isolation rates of Escherichia coli, ESBL, and AmpC among patients at a hospital in Tigray, Northern Ethiopia Yes: denotes *E. coli* isolated; No: indicates absence. M: male; F: female; B: blood; P: Pus; U: urine. TOS: type of specimen; COM: comorbidities; HAM: history of antimicrobial Use; IDM: indwelling medical device; LOH: length of hospitalization (in days); ESBL: extended-spectrum beta-lactamase; AmpC: ampicillin C beta-lactamase; MW: medical ward; OPD: outpatient department; PW: pediatric ward; SW: surgical ward

Variables		Isolation of *E. coli* in different wards								Total number of isolates (%)	X^2^(p-value)	ESBL-AmpC carrying *E. coli* isolates in different wards								X^2^(P-value)
		MW		SW		PW		OPD				MW		SW		PW		OPD		
		Yes	No	Yes	No	Yes	No	Yes	No			ESBL	AmpC	ESBL	AmpC	ESBL	AmpC	ESBL	AmpC	
Age range	<18	6	1	1	3	26	17	3	2	36 (33)	90.1 (0.000)	2	2	0	0	13	16	2	2	90 (0.000)
	19-39	18	61	10	23	1	22	8	26	37 (34)		6	7	1	2	0	1	2	2	
	40-59	20	36	2	14	0	0	6	31	28 (25.7)		12	12	2	2	0	0	2	2	
	>60	5	36	3	7	0	0	0	29	8 (7.3)		3	4	3	3	0	0	0	0	
	Total	49	134	16	47	27	39	17	88	109 (100)		23	25	10	11	13	17	5	6	
Sex	M	23	62	7	25	13	16	10	37	53 (48.62)	8.81 (0.032)	11	12	5	5	7	9	3	4	0.301 (0.96)
	F	26	72	9	22	14	23	7	51	56 (51.38)		12	13	5	6	6	8	2	2	
	Total	49	134	16	47	27	39	17	88	109		23	25	10	11	13	17	5	6	
TOS	B	3	29	1	5	9	20	1	16	14 (12.84)	16.468 (0.001)	1	1	1	1	3	5	1	1	10.963 (0.09)
	P	13	16	10	38	4	10	1	16	28 (25.69)		5	7	6	7	4	4	0	0	
	U	33	89	5	4	14	9	15	56	67 (61.46)		17	17	3	3	6	8	4	5	
	Total	49	134	16	47	27	39	17	88	109		23	25	10	11	13	17	5	6	
COM	Yes	26	47	1	27	14	3	1	21	42 (38.53)	8.53 (0.036)	13	14	1	1	8	8	1	1	8.88 (0.031)
	No	23	87	15	20	13	36	16	67	67 (61.46)		10	11	9	10	5	9	4	5	
	Total	49	134	16	47	27	39	17	88	109		23	25	10	11	13	17	5	6	
HAM	Yes	18	82	13	16	16	22	6	22	53 (48.62)	13.56 (0.004)	10	11	9	10	10	12	4	5	8.7 (0.034)
	No	31	52	3	31	11	17	11	66	56 (51.38)		13	14	1	1	3	5	1	1	
	Total	49	134	16	47	27	39	17	88	109		23	25	10	11	13	17	5	6	
IMD	Yes	31	50	7	33	18	24	2	14	58 (53.21)	9.935 (0.019)	17	18	5	6	10	13	1	1	7.026 (0.071)
	No	18	84	9	14	9	15	15	74	51 (46.79)		6	7	5	5	3	4	4	5	
	Total	49	134	16	47	27	39	17	88	109		23	25	10	11	13	17	5	6	
LOH (days)	0	5	0	1	0	1	0	17	88	24 (22.01)	31.695 (0.000)	0	0	0	0	0	0	5	6	91.08 (0.012)
	1-7	4	85	3	24	12	25		-	19 (17.43)		7	8	3	4	5	4	0	1	
	>8	40	49	12	23	14	14		-	66 (60.55)		16	17	7	7	8	13	0	0	
	Total	49	134	16	47	27	39	17	88	109 (26.13)		23	25	10	11	13	17	5	6	

Antimicrobial resistance profile

**Figure 1:**
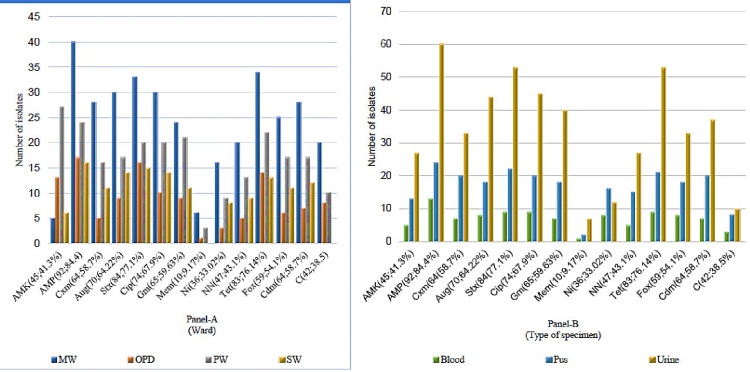
E. coli antimicrobial resistance patterns by ward (Panel A) and specimen type (Panel B) at a hospital in Tigray, Northern Ethiopia. MW: medical ward; OPD: outpatient department; PW: pediatric ward; SW: surgical ward; Amk: amikacin; Amp: ampicillin; Cxm: cefotaxime; Aug: amoxicillin + clavulanic acid; STX: sulfamethoxazole + trimethoprim; Cip: ciprofloxacin; Gm: gentamicin; Mem: meropenem; Ni: nitrofurantoin; NN: tobramycin; Tet: tetracycline; Fox: cefoxitin; Cdm: ceftazidime; C: chloramphenicol. Panel A presents the percentage distribution of antimicrobial resistance across hospital wards, while Panel B displays the distribution of antimicrobial resistance across various specimen types

The *E. coli* isolates exhibited varying resistance levels to the antibiotics tested. The highest rates of resistance were found to ampicillin (92 isolates; 84.4%) and trimethoprim-sulfamethoxazole (84 isolates; 77%). Moderate resistance was observed to ciprofloxacin (74 isolates; 68%) and cefotaxime (64 isolates; 58.7%). Nitrofurantoin (36 isolates; 33%) and meropenem (10 isolates; 9.17%) showed relatively low resistance levels. Notably, only 7 isolates (6.4%) remained susceptible to all antibiotics tested, while 9 isolates (8.3%) were resistant to every drug examined (Figure 1).

**Figure 2:**
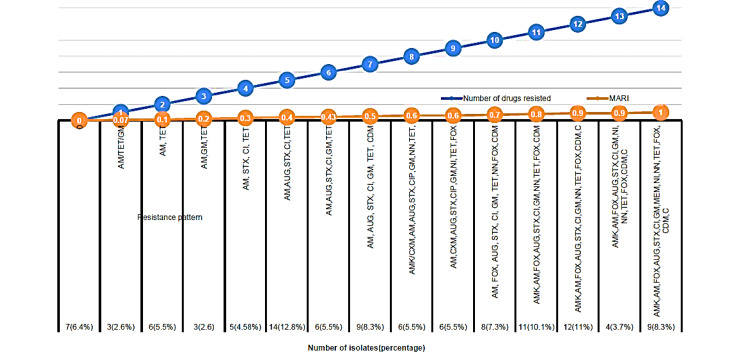
Distribution of E. coli isolates by resistance patterns, drug resistance levels, and multiple antibiotic resistance index (MARI) at a Hospital in Tigray, Northern Ethiopia Amk: amikacin; Amp: ampicillin; Cxm: cefotaxime; Aug: amoxicillin + clavulanic acid; STX: sulfamethoxazole + trimethoprim; Cip: ciprofloxacin; Gm: gentamicin; Mem: meropenem; Ni: nitrofurantoin; NN: tobramycin; Tet: tetracycline; Fox: cefoxitin; Cdm: ceftazidime; C: chloramphenicol; MARI: multiple antibiotic resistance index

The MAR index ranged from 0.07 (for an isolate resistant to ampicillin, tetracycline, and gentamicin) to 1.00 (for an isolate resistant to all antimicrobials). MARI of >0.2 and MDR was observed in 93 (85.3%) of isolates. A higher MARI value indicates a greater degree of multidrug resistance (Figure 2).

ESBL and AmpC-production

51 (46.8%) of the isolates were found to carry ESBL, with carriage being higher in the medical ward (23, 45.1%) and lower in the outpatient department (5, 9.8%). Moreover, 59 isolates (54%) displayed AmpC beta-lactamase activity, with a higher prevalence observed in the medical ward (25 isolates, 42.4%). The study also confirmed that 48 (44%) isolates exhibited the presence of both ESBL and AmpC production in certain isolates, indicating multiple resistance mechanisms. A statistically significant association was observed between ESBL, AmpC carriage and comorbidities (p=0.031), history of antimicrobial use (p=0.034), and length of hospital stay (p=0.012) (Table 2).

Biofilm formation and its determinants

**Figure 3:**
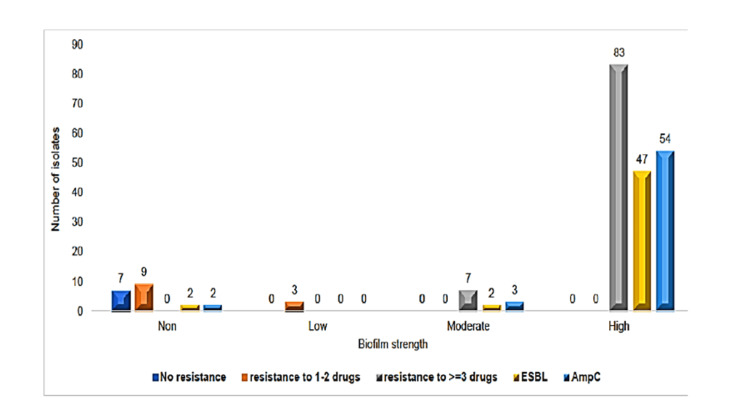
Distribution of E. coli isolates by biofilm strength and associated drug resistance levels *non: non-biofilm formers; low: low biofilm formers; moderate: moderate biofilm formers; high: high biofilm formers; ESBL: extended-spectrum beta-lactamase; AmpC: Ampicillin C

The TCP showed 83 out of the 109 *E. coli* isolates as high biofilm producers, and three of the remaining isolates were categorized as low biofilm formers(Table 3). Among biofilm-forming isolates, 52/83 (62.65%) were distributed in urine specimens, and medical ward accounts for the highest proportion with counts of strong virulent biofilm formers; 38/83(45.78%). It is interesting to find that 47 isolates with ESBL and 54 isolates with AmpC also produced high biofilm (Figure 3).

Biofilm formation was also significantly associated with the use of indwelling medical devices, most notably catheters (p=0.0001), as 30/35 isolates isolated from organisms adhering to catheters were high biofilm producers, representing an overall percentage of 85.7%. Isolates from IV lines, in contrast, demonstrated an equal distribution of more *E.coli, *and the category ‘others’ showed 75% (3/4) as high producers. Furthermore, 56.4% (22/39) of isolates without indwelling devices were high biofilm producers. Multiple regression analysis revealed that biofilm formation ability was significantly related to antimicrobial history (p=0.011), ward (p<0.001), indwelling device use(p<0.000), and hospitalization length in days (p<0.000) (Table 3). As a limitation, the TCP assay for biofilm detection used crystal violet staining that could overestimate adherent bacteria, although additional standards to reduce interference and improve result accuracy were employed as a mitigation strategy.

**Table 3:** Rates of antimicrobial resistance in E. coli isolates by biofilm strength and optical density at a hospital in Tigray, Northern Ethiopia MW: medical ward, OPD: outpatient department, PW: pediatric ward, SW: surgical ward; no: indicates the absence of clinical factor, yes: indicates presence; non: refers to non-biofilm forming isolates, low: to low biofilm-forming isolates, moderate: indicates to moderate biofilm-forming isolates, and high: indicates to high biofilm-forming isolates, each followed by their respective optical densities in parentheses. Optical density is dimensionless. IV-line: intravenous line.

Variables	Number of isolates exhibiting varying strengths of biofilm formation (optical density)				
Ward	Non (<0.11)	Low (>0.11)	Moderate (0.1234-0.1271)	High (0.1287-0.3984)	Chi-square (p-value)
MW	11	0	6	32	28.735 (0.001)
OPD	1	0	0	16	
PW	3	3	1	21	
SW	1	0	0	14	
Total	16	3	7	83	
Age range					
<18	4	3	2	27	90.14 (0.000)
19-39	3	0	4	30	
40-59	5	0	1	22	
>60	4	0	0	4	
Total	16	3	7	83	
Comorbidities					
No	11	1	3	52	3.552 (0.314)
Yes	5	2	4	31	
Total	16	3	7	83	
Type of specimen					
Blood	2	1	2	9	11.011 (0.088)
Pus	6	0	0	22	
Urine	8	2	5	52	
Total	16	3	7	83	
History of antimicrobial use					
Yes	6	2	3	42	11.122 (0.011)
No	10	1	4	41	
Total	16	3	7	83	
Use of indwelling medical devices					
Yes	4	3	2	44	19.571 (0.000)
Catheter	1	2	2	35	
IV-line	2	1	0	5	
Others	1	0	0	4	
No	12	0	5	39	
Total	16	3	7	83	
Sex					
Male	2	3	6	42	4.956 (0.175)
Female	14	0	1	41	
Total	16	3	7	83	
Length of hospitalization in days					
0	4	0	0	20	251.401 (0.000)
1-7	1	2	3	13	
>8	11	1	4	50	
Total	16 (14.7%)	3 (2.8%)	7 (6.42%)	83 (76.15%)	

## Discussion

The present study investigated the drug-resistance patterns and biofilm-forming traits of *E. coli* clinical isolates from Tigray, Northern Ethiopia. 109 isolates of *E. coli,* totaling 26.14% of all analyzed samples, were identified, showing that this pathogen is relevant to various infections in our region.

Compared to an earlier study from Ethiopia, when our findings were compared, 54.7% of *E. coli* recovered less than a subsequent Ethiopian report [13]. While these differences might be due to different study populations or sampling methods and infection controls in place. On the other hand, our recovery rate is lower compared to those reported from Addis Ababa (12.4%)[18-19] and Ghana(15.76%) [20]. These variations in recovery rates could be due to differences in infection prevalence geographically and by demographics, as well as inequities in access to healthcare between regions.

A substantial percentage of the *E. coli* isolates in this study were found to be resistant to ampicillin (84.4%), followed by trimethoprim-sulfamethoxazole (77.1%). The least resistance was observed against meropenem, 9.17%. The resistance rates are lower than in the study conducted at Tikur Anbesa Specialized Hospital, which reported that 94.6% of isolates were ampicillin-resistant [21]. A separate study from Egypt revealed slightly lower resistance rates for ampicillin at 88%, trimethoprim-sulfamethoxazole at 63%, and meropenem at merely 3 %[22]. While the rate of resistance here varied between studies, the increasing trend in *E. coli *rates of common and switch-on antibiotics is concerning as these are likely to be both prescribed for HCA UTI and have significant implications on patient outcomes.

We found an MDR pattern in 85.3% of the *E.coli* isolates with multiple antibiotic resistances or MAR index of more than 0.2 This result reveals the difficulties in managing infections due to such isolates since a larger MAR index means more multi-drug resistant. The figure is higher than MDR rates reported in earlier Ethiopian studies, which varied between 27% and 58% [21,23]. This could be related to changes in patient movements across borders now carrying drug-resistant isolates, as well as an absence of good infection control practices.

Additionally, we observed that 48.6% of the *E coli* isolates possessed Extended-Spectrum Beta-Lactamases (ESBL), and 54% carried AmpC enzymes. This was higher than the 1.2% and 44.6%, respectively, reported in prior studies from Addis Ababa [24]. However, our results were lower than those of a study in another area of Ethiopia, which reported an ESBL carriage rate of 54.9% [25], and ESBL carrying in Iran showed a prevalence rate of 41.7% [15]. The higher proportion of *E. coli* isolates producing ESBL and AmpC in our study may be attributable to the overuse of broad-spectrum antibiotics for empirical treatment in this area.

Moreover, we found that 85.3% of the *E. coli *isolates were biofilm producers, which was higher than that of a previous study in Jimma, Ethiopia [23]. Moreover, our study revealed that *E. coli* isolates with biofilm formation potential were more resistant to conventional antimicrobial agents than non-biofilm formers as well. Modernize situates 76.16% of the computed MDR isolates as dominating biofilm former, acting just moderate size (6.4%). These trends correlate with a prior study conducted in Uganda, where it was found that 64% of MDR isolates were capable of biofilm formation [26].

These above results underscore the important relationship between biofilm formation and antimicrobial resistance among *E. coli* isolates, in a particular form of clinical relevance regarding infections associated with a biofilm mode growth pattern. The high rate of biofilm-producing MDR isolates indicates that it is important to develop a new approach against such resistant strains, and this could include strategies targeting the ability to form biofilms in them.

Our findings also demonstrated a high positive correlation of antimicrobial consumption leading to significant increased rates of AMR in *E. coli *isolates, especially among patients with indwelling devices, history of prior antibiotic use and prolonged duration of hospitalization. More than half (52.7%) of biofilm-producing *E. coli* isolates in this study were from patients with indwelling medical devices. This aligns with previous studies [23,27]. This increased resistance may be because drug-resistant pathogens hide in biofilms on these implanted medical devices. Biofilm matrix hinders antimicrobial penetration, thus providing a habitat where such pathogens can survive and possibly promoting the induction of efflux pumps/genetic determinants as the length of hospital stay increases [6, 28-30]. The frequency of MDR biofilm producers in patients with indwelling devices further demonstrates the challenges associated with biofilm-mediated infections within a clinical context. This report underscores the importance of a comprehensive infection control strategy and ongoing monitoring for antimicrobial stewardship in patients requiring long-term device support.

## Conclusions

Although carbapenems are the most effective antimicrobials for treating *E. coli* infections, the increasing number of resistant isolates underscores the urgent need to tackle antimicrobial resistance. Future strategies should emphasize the development of new anti-biofilm agents based on our findings, along with implementing effective sterilization and disinfection protocols for medical instruments and devices to mitigate the spread of resistant isolates.
